# Enhancing Diagnostic Decision-Making: Ensemble Learning Techniques for Reliable Stress Level Classification

**DOI:** 10.3390/diagnostics13223455

**Published:** 2023-11-16

**Authors:** Raghav V. Anand, Abdul Quadir Md, Shabana Urooj, Senthilkumar Mohan, Mohamad A. Alawad, Adittya C.

**Affiliations:** 1 School of Computer Science and Engineering, Vellore Institute of Technology, Chennai 600127, India; raghavanand.v2019@vitstudent.ac.in (R.V.A.); adittya.c2019@vitstudent.ac.in (A.C.); 2 Department of Electrical Engineering, College of Engineering, Princess Nourah bint Abdulrahman University, P.O. Box 84428, Riyadh 11671, Saudi Arabia; 3 School of Information Technology and Engineering, Vellore Institute of Technology, Vellore 632014, India; senthilkumar.mohan@vit.ac.in; 4 Department of Electrical Engineering, Imam Mohammad Ibn Saud Islamic University, Riyadh 11432, Saudi Arabia; maawaad@imamu.edu.sa

**Keywords:** stress, students, academic, routine, mitigate, tension, conventional, boosting, metrics, practical analysis, model, performance

## Abstract

An intense level of academic pressure causes students to experience stress, and if this stress is not addressed, it can cause adverse mental and physical effects. Since the pandemic situation, students have received more assignments and other tasks due to the shift of classes to an online mode. Students may not realize that they are stressed, but it may be evident from other factors, including sleep deprivation and changes in eating habits. In this context, this paper presents a novel ensemble learning approach that proposes an architecture for stress level classification. It analyzes certain factors such as the sleep hours, productive time periods, screen time, weekly assignments and their submission statuses, and the studying methodology that contribute to stress among the students by collecting a survey from the student community. The survey data are preprocessed to categorize stress levels into three categories: highly stressed, manageable stress, and no stress. For the analysis of the minority class, oversampling methodology is used to remove the imbalance in the dataset, and decision tree, random forest classifier, AdaBoost, gradient boost, and ensemble learning algorithms with various combinations are implemented. To assess the model’s performance, different metrics were used, such as the confusion matrix, accuracy, precision, recall, and F1 score. The results showed that the efficient ensemble learning academic stress classifier gave an accuracy of 93.48% and an F1 score of 93.14%. Fivefold cross-validation was also performed, and an accuracy of 93.45% was achieved. The receiver operating characteristic curve (ROC) value gave an accuracy of 98% for the no-stress category, while providing a 91% true positive rate for manageable and high-stress classes. The proposed ensemble learning with fivefold cross-validation outperformed various state-of-the-art algorithms to predict the stress level accurately. By using these results, students can identify areas for improvement, thereby reducing their stress levels and altering their academic lifestyles, thereby making our stress prediction approach more effective.

## 1. Introduction

The pandemic has played its role to keep classes for students online, and it has significantly increased the stress levels of the students because they must look at devices such as laptops, PCs (personal computers), and mobiles throughout the day, complete assignments, and handle exams online. This is the primary reason for an increase in stress amongst college students in the United States [1]. This physically affects the students because they sit in one place, and there is less physical activity compared to how it would have been if the classes were conducted in schools and colleges on campus [2]. To identify certain academic factors that affect students, a survey was conducted across various schools and colleges about their workloads and collected their responses. With the use of machine learning models, which play a significant role in recognizing patterns that are not visible to the naked eye, this paper aims to predict the stress levels of the students based on the responses collected from them.

There are many factors that contribute to stress such as personal relationships, worrying about something, uncertainty in taking decisions, and so on [3], but the focus here will be on the academic factors that contribute to the stress. After brainstorming various ideas, certain key features were picked that may contribute to academic stress, and those questions were asked of students in the teenage group, and the responses were collected along with the stress levels, which will be used as the training data for the purpose of predicting stress levels for other students in future using the model. The model thus provides insights to the students to find the ideal habits that they need to follow in day-to-day life in order to keep their stress levels under control. Machine learning models such as the decision tree classification algorithm, random forest algorithm (bagging), AdaBoost algorithm, and gradient boosting algorithm have been developed on the collected data to obtain various insights, thus maximizing the efficiency of the output for predicting stress levels and determining a student’s ideal workload and daily habits to live a stress-free student life. A challenge faced before building the model was the bias in the collected survey. The students who responded to the survey mostly responded that their stress levels were either in a manageable state or that they were highly stressed; hence, the numbers of responses for the no-stress category were fewer, which in turn caused an imbalance in the dataset. In order to solve this issue, the oversampling method was used to bring the count of the minority class, that is, the no-stress responses, to the other two majority classes. This ensured that there was no bias in the trained machine learning models that would be used to predict the stress levels on the testing data.

To overcome the challenges, the key contributions involved in the paper are as follows:

(i) The research proposes a unique ensemble learning strategy designed primarily for classifying students’ levels of stress. While earlier research focused on stress prediction as outlined in Section 2, the work presented here ingeniously blends decision trees, random forest classifiers, AdaBoost, gradient boost, and ensemble learning techniques to provide a thorough model for precisely classifying stress levels.
(ii) In contrast to other approaches outlined in Section 2, the article incorporates survey data from the student population to thoroughly analyze stress causes. The method also uses oversampling methods to correct the imbalance in the dataset of stress levels. A more reliable and accurate stress prediction model is produced because of the integration of survey-based insights with imbalance management.
(iii) The work uses a number of performance assessment criteria, such as accuracy, precision, recall, and F1 score, to go beyond intuitive measurements. The results’ validity is further strengthened by the use of fivefold cross-validation. The addition of receiver operating characteristic (ROC) curve analysis also offers a full review of the model’s performance for each category of stress level, demonstrating a comprehensive and in-depth examination of the suggested strategy.

Bagging and boosting techniques have been used to increase the efficiency of the predictions. The objective of boosting is to combine weak learners together to form an overall strong model. However, it is important to note that bagging is computationally more effective than boosting because it can train ‘n’ models in parallel, while boosting cannot do that. If we do not have a high bias and there is only a need to reduce overfitting, bagging was the best option to choose, which was another challenge that had to be overcome for the analysis of the survey data.

Academics play an important role in determining the future of a student, and this can cause mental pressure by testing their coping skills [4]. Keeping this in mind, management of stress plays a crucial role while navigating through challenges faced by the students in their academic coping lifestyles. The COVID-19 pandemic caused many struggles in a student’s life when classes and assignments were given in online modes [5]. This caused an increase in screentime and working hours, thereby imposing more pressure on the students. Thus, this paper aims to analyze which factors affect the way students cope with academics by obtaining responses from them and building a model that can predict how well they manage their stress levels. This enables them to adapt and improvise their lifestyles to keep their stress levels in check.

The Section 2 of the paper talks about the various related work conducted in the field of stress prediction and the models used for that purpose; the Section 3 explains the proposed work and the models used to obtain the results, followed by the Section 4 and Section 5 that explain the experimental setup and the results obtained in comparison with the other works, with a summary of the various performance metrics that determine the efficiency of the model.

## 2. Related Works

In this section, related papers are referenced and surveyed. It is noted that different authors used different methodologies in implementing their solutions to similar stress-level balancing problems. Some used deep learning using neural networks and long short-term memory (LSTM) models, while many used supervised learning classification methods such as random forests, decision trees, and other ensemble learning algorithms.

### 2.1. Deep Learning Algorithms

The authors in [6] tried to predict stress levels using LSTM. The dataset is obtained from a student life app, which collects passive and automatic data from the sensors available in mobiles. The data were collected from students of Dartmouth for a 10-week spring term. They concluded that they were 62.83% confident in determining if the student was stressed or not by observing their last 2–12 h of usage of their mobiles. They stated that if the number of epochs were increased, the training accuracy reached a maximum of 99%, but a drop in the performance of the testing set was noticed. The authors in [7,8,9,10,11,12] proposed a method to predict personalized stress in students using a deep multitask network. They received the dataset from a SmartLife study of 48 students in Dartmouth. They concluded that the best model that predicted stress levels accurately was the CALM network model with an F1 score of 0.594.

### 2.2. Supervised and Ensemble Learning

There are works that address a similar problem to ours, and the authors of those research projects use different methods to arrive at a conclusion for their specific problems. Some used deep learning algorithms to classify the data they collected. Others used various machine learning algorithms such as ensemble algorithms, boosting algorithms, etc. In [10,11,12], the authors took the Open Sourcing Mental Illness (OSMI) Mental Health in Tech 2017 survey as their dataset. They initially performed one-hot encoding after cleaning the data, and they took 14 of the most relevant attributes out of 68. One-hot encoding is a popular encoding method to utilize when processing datasets containing categorical variables [13]. They finally concluded that stress is highly correlated to gender among the selected parameters, i.e., women are generally found to experience greater stress than men in the same department. The authors in [14] researched about detecting anxiety, depression, and stress using the dataset obtained from the Depression, Anxiety, and Stress Scale (DASS 21) questionnaire. Their results show that the highest accuracy for all three classes, depression, anxiety, and stress, is achieved in the naïve Bayes classifier; however, the F1 score for stress was highest in the random forest algorithm, and for depression, it was highest in the naïve Bayes classifier with the F1 score for anxiety being low in all tested models. They also stated that the patients without diseases, i.e., negative cases, were also classified appropriately. In [15], the authors researched the prevalence and the predicting causes of stress among university students in Bangladesh. They surveyed students from 28 different universities using questions regarding academic-, health-, and lifestyle-related information, which in turn was referred to the perceived stress status of the students. They stated that the most important factors that were selected for the prediction of stress were academic background, blood pressure, pulse rate, sleep status, etc. The highest performance was observed from the random forest algorithm with an accuracy of nearly 80%, and the lowest was observed by logistic regression with an accuracy of 75%. They concluded that their model predicts the psychological problem of students more accurately, and this can help stakeholders, families, management, and authorities to understand the health problems faced by the students.

The authors in [16] tried to model students’ happiness by using machine learning methods on data collected from students who were monitored for a month via mobile phone logs and sensors. They stated that modeling and predicting the happiness of the students can help to detect individuals who are either prone to or at risk of depression, and then, they can intervene and help the student.

They finally stated that they had achieved 70% classification accuracy of the happiness of students on the test data. In [17,18], perceived stress caused by the COVID-19 pandemic on adults were modeled and predicted through machine learning models and psychological traits. They collected data from around 2000 Italian adults via online surveying methods concerning their stress factors, psychological traits, demographics, etc. They stated that higher levels of distress were observed in the parts of society where people earn less. It was also found that women were comparatively more stressed, and those who lived with others also faced more distress. They finally said that the machine learning models identified people with high stress with a sensitivity of more than 76%.

The authors in [19,20] tried to predict stress in the students who are transitioning from teenagers to adults in a few institutions of India, and the data were collected from them. The top-most contributors of stress in those people were found to be social media, academic pressure, workload, and anxiety, among others. The data showed that B. Tech students are under more high stress as compared to bachelor of computer application (BCA) students. Their research also showed that academics, work stress, and unhealthy social media consumption contributed much toward stress among generation Z students. Finally, their algorithm resulted in an R-squared value of 0.8042 after elimination of around 30% of the initial features obtained. In [21], the authors tried to detect and predict high-resolution stress as a tool for electronic or mobile health systems supporting personalized treatments both clinically and remotely. The dataset they used to train their models was the Wearable Stress and Affect Detection (WESAD) dataset, and they calculated stress scores based on various questionnaires from it. Their results show that the specific algorithms of random forests, least-squares gradient boosting, and nonlinear auto-regressive network with exogenous inputs offered the best predictions of high-resolution stress, and they also proposed that this can be integrated with a decision support system to aid in the decision-making for stress management and monitoring.

### 2.3. Analysis of Stress Factors

In [22,23], the authors tried to find the interrelationship between stressors, i.e., stress-causing agents, and coping strategies. They used self-collected data from various students from five colleges in the city of Shillong. They reported that academic stress had a high degree of correlation with social and financial stress, and positive stress-coping mechanisms such as prayer, sleep, and meditation were helpful to combat academic stress. The authors in [24] tried to predict stress in pre-registration nursing students using self-collected data from all the pre-registration nursing students in a top English university. They finally stated that the work–life balance of pre-registration nursing students, especially those who must take care of children, should be considered important while designing the curriculum of nursing education. They also said that the main predictors of caseness are pressure, whether they had children or not, coping method employed, and scores on their personal problems, and the caseness rate was around 33% of the population. Table 1 shows the summary of the key contributions and algorithms used in the various surveyed works.

In [11], the authors discussed that the global pandemic was a cause of fear and stress being instigated among the people, with students especially becoming stressed over their studies in various forms. Their analysis showed that there was a key importance in certain features affecting academic stress such as prolonged use of digital tools for education purposes, lack of physical learning on campus contributing to improper education, and the psychological elements at play. In [1], the authors surveyed students from Texas A&M University regarding stress. Over 40% of surveyed students reported an increase in stress due to online classes and then, concerns over grades. Participants had difficulty concentrating and had an increased workload that contributed to their stress. Some also stated that their sleep schedules have also been impacted, causing stress. In [25], the authors said that students suddenly feel less motivated to focus on academics due to the sudden switch from traditional teaching to an online mode. Students also believe they are wasting a lot of time indulging in social media as they do not have anything interesting to do and as a diversion from academia.

Four stress parameters are taken into consideration in distinguishing each work analyzed, namely academic/student-related stress (S1), workplace-related stress (S2), personal stress (S3), and stress caused by the COVID-19 pandemic (S4). For each of the authors mentioned below in Table 2, the stress factors they have considered in their work are represented.

The various works related to the management and prediction of stress were surveyed and analyzed. A standard benchmark for the prediction of stress levels caused by the impact of academics is proposed and implemented in this paper with this trend in mind.

## 3. Problem Statement

The primary focus of the problem is to classify the students’ stress levels and classify them into three categories: whether they are extremely stressed, experiencing manageable stress, or are completely free from any mental pressure brought on by analyzing particular academic factors. Ensemble learning models such as random forest and boosting algorithms such as the AdaBoost and gradient boost have been utilized after a traditional approach such as the decision tree classifier to determine if there is any improvement in the efficacy of the findings so acquired. The data used for this purpose were collected from the student community using a survey questionnaire. The attributes of the model and the methods used to construct it are described in the following sections.

## 4. Proposed Work

**Figure 1:**
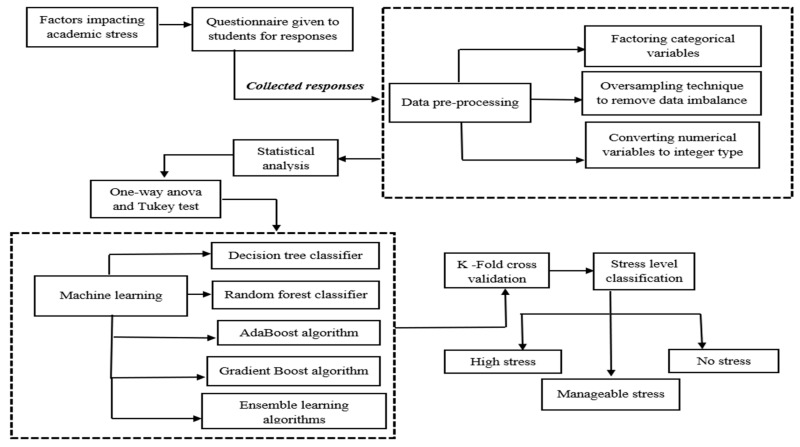
Architectural workflow for academic stress level classification.

After collecting the survey data, preprocessing work and various machine learning models are applied to classify the stress levels into three categories: highly stressed, manageable stress, and no stress. The oversampling method is used to remove the imbalance in the dataset caused by the minority class. Decision tree, random forest classification, AdaBoost, and the gradient boost algorithm are used as the ML models for the analysis. Figure 1 represents the architecture of the entire process that is followed.

The oversampling methodology used to balance the dataset is described in Algorithm 1.
**Algorithm 1:** Oversampling the minority class in survey data to remove biasStep 1The minority class, “No stress” class, is oversampled to remove bias.Step 2Oversampling the data due to the imbalance in the dataset for the “No stress” class. No stress data ← take subset (data, where stress level = “No stress”) Sampling “no stress” subset data to generate duplicate rows ← Sample ‘n’ rows (no-stress data, specify additional data point count)Step 3Merge the additional duplicate records created with the initial data        New data ← Row binding the data frame (data, Sampled “no stress” data)        Shuffled data ← new data (sample (1: total data point count of new obtained data))Step 4Use the obtained data to train machine learning models

The algorithm takes the imbalanced dataset and adds more samples to replicate the “No stress” class, and it is merged with the initial dataset to form the data that will be provided as input to the models.

### 4.1. Decision Tree Classification to Classify Stress Levels

Decision trees use the concept of entropy and information gain to build trees from a root node. Entropy is defined as the measure of randomness of a variable, while the information gain computes the difference between the entropy value before and after the splitting of the tree and specifies the impurity in the class elements [26]. Here, the stress levels are classified by using both criteria to extract information from the dataset.

The entropy of an attribute is calculated by using Equation (1).

(1) $$\;E(S)=\sum_{i=1}^{c}\;-p_{i}{log}_{2}p_{i}$$

where *E(S)* is the entropy of attribute *S*, and *p_i_* is the probability of event i or the percentage of class *i* in a node of *S*.

Each node in the tree yields a maximum amount of data in each split, which could be achieved using the information gain (*IG*) provided by Equation (2).

(2) $$\;\;\;\;\;\;\;\;\;\;\;\;\;\begin{array}{c} IG\left(D_{p},f\right)=I\left(D_{p}\right)-\sum_{j=1}^{m}\;\;\frac{N_{j}}{N_{p}}I\left(D_{j}\right)\\ IG\left(D_{p},a\right)=I\left(D_{p}\right)-\frac{N_{left}}{N_{p}}I\left(D_{left}\right)-\frac{N_{\mathrm{right}\;}}{N_{p}}I\left(D_{\mathrm{right}\;}\right) \end{array}\;\;\;$$

The parameters used to train the decision tree classifier are described in Algorithm 2.
**Algorithm 2:** Implementing decision tree classifier to classify stress levelsStep 1Read the surveyed datasetStep 2Transforming data to feed them to the machine learning model Data ← transform (data, sleep = convert as integer (sleep), productivity ← convert as factor (productivity), screentime ← convert as factor (screentime), assignments ← convert as integer (assignments), deadline ← convert as factor(deadline), study ← convert as factor (study), stress ← convert as factor (stress))Step 3Training the decision tree model using the unbiased dataset Sample ← sample rows (1: total data point count (data), split into 80–20 ratio) Train ← data[sample,] Test ← data[-sample,] Decision tree model ← feed data (stress ~., data = training data)Step 4Interpreting the results

The algorithm takes the dataset as the input and converts the categorical variables such as the productivity and study-time columns into factors and the assignment column into an integer, which act as the preprocessing steps before splitting the training and testing data and feeding them into the decision tree classifier.

### 4.2. Random Forest Classification to Classify Stress Levels

The random forest algorithm is an ensemble learning method that uses the concept of bagging. The random forests select a subset of the features in the survey dataset, and the final classification of the stress levels is obtained by training the model using multiple decision trees as base learners [27]. It uses the concepts of the Gini index and entropy, which are calculated using Equations (3) and (4).

(3) $$\mathrm{Gini}=1-\;\sum_{i=1}^{c}{\left(p_{i}\right)}^{2}\;\;$$

(4) $$\mathrm{Entropy}=\sum_{i=1}^{C}\;-p_{i}*{log}_{2}\left(p_{i}\right)$$

where *p_i_* is the probability of event *i* or the percentage of class *i* in a node of *S*.

The parameters used to train the random forest model are described in Algorithm 3.
**Algorithm 3:** Feeding the data to random forest classifierStep 1Implement the similar transformation steps used in decision tree classifierStep 2Random forest model ← random forest (stress~., data = training data, mtry = 2)Step 3Evaluate the model using performance metrics Step 4Interpreting the results

The algorithm follows the preprocessing steps described in the previous algorithm and feeds the data to the random forest classifier.

### 4.3. Stress Level Classification Using Adaboost Algorithm

This is another ensemble learning technique that uses the concept of boosting [28]. Decision tree “stumps” are used as base learners, and each time a wrong classification is made on the base learners, those weak links are alone passed to the next stump, and this process keeps happening until the error is minimized. Weights are assigned to all the data points, and after each time a wrong classification happens, higher weights are assigned to those points.

Equation (5) calculates the weights of the data points.

(5) $$w\left(x_{i}y_{i}\right)=\frac{1}{N},i=1,2,\ldots n$$

where *n* is the total number of data points.

The performance of each stump is calculated by the formula in Equation (6).

(6) $$\mathrm{Performance}\;\mathrm{of}\;\mathrm{the}\;\mathrm{stump}=\frac{1}{2}{log}_{e}\left(\frac{1-\mathrm{Total}\;\mathrm{Error}}{\mathrm{Total}\;\mathrm{Error}}\right)$$

Then, the new weights after each iteration are updated using Equation (7).

(7) $$\mathrm{New}\;\mathrm{weight}=\;\mathrm{Old}\;\mathrm{Weight}\;*\;e^{\pm \left(\mathrm{Performance}\right)}$$

The parameters used to train the AdaBoost model are described in Algorithm 4.
**Algorithm 4:** Feeding the data to the AdaBoost classifier**Step 1**Implement the similar transformation steps used in decision tree classifier**Step 2**Model ← boosting (stress~., data = training data, boost = TRUE, mfinal = 100) predictions ← predict (model, test)**Step 3**Evaluate the model using performance metrics **Step 4**Interpreting the results

The algorithm follows the preprocessing steps described in the previous algorithm and feeds the data to the AdaBoost algorithm with the parameters mentioned above.

### 4.4. Stress Level Classification Using Gradient Boost Algorithm

The gradient boost algorithm is another boosting technique under the ensemble learning method. It also uses the concept of decision stumps. It increases the weight of the records that are incorrectly classified and decreases the weight of the ones that are correctly classified. It works on the principle that the weak learners combine together to form a strong model [29]. It is described in Equation (8).

(8) $$F_{0}(x)=argmin\sum_{i=1}^{n}\;L\left(y_{i},\gamma \right)$$

where *y_i_* is the observed value of each observation, *L* is the loss function, and gamma is the value for log (odds).

The derivative of the loss function is provided by Equation (9).

(9) $$\frac{d}{\mathrm{dlog}\left(odds\right)}obs\;*\;\log\left(odds\right)+\log\left(1+e^{\log(odds)}\right)$$

The gamma value that minimizes the loss function can be written in a generalized equation, as below:

(10) $$\gamma =\frac{\mathrm{Sum}\;\mathrm{of}\;\mathrm{residuals}}{\mathrm{Sum}\;\mathrm{of}\;\mathrm{each}\;p(1-p)\;\mathrm{for}\;\mathrm{each}\;\mathrm{sample}\;\mathrm{in}\;\mathrm{the}\;\mathrm{leaf}}$$

And finally, the predictions are updated using Equation (11).

(11) $$F_{m}(x)=F_{m-1}(x)+\nu \sum_{j=1}^{J_{m}}\;\gamma_{jm}I\left(x\in R_{jm}\right)$$

The parameters used to train the gradient boost algorithm are described in Algorithm 5.
**Algorithm 5:** Feeding the data to gradient boost classifierStep 1Implement the similar transformation steps used in decision tree classifierStep 2Model gbm = gbm (stress~., data = training_data, distribution = “multinomial”, cv.folds = 10, shrinkage = 0.01, n.minobsinnode = 10, n.trees = 200)Step 3Evaluate the model using performance metrics Step 4Interpreting the results

The algorithm follows the preprocessing steps described in the previous algorithm and feeds the data to the gradient boost algorithm with the parameters mentioned above, and the combinations of the above algorithms are analyzed for ensemble learning. The next section describes the exploratory analysis of the factors involved in determining the academic stress level of students and the application of these algorithms with the results obtained.

## 5. Experimental Setup and Analysis

### 5.1. Exploratory Analysis

The survey data were collected from the student community in the teenage group, and the experiments were conducted using the RStudio environment, version 1.4.1717-3. R-programming version 4.1.1 was used to do the preprocessing and the building of machine learning models.

The following questions were the parts of the survey that were answered by the students and were used as the dataset for training the models:

- Number of hours of sleep every night;
- Most productive time in the day—early bird/night owl;
- Screen time per day;
- Number of weekly assignments assigned to the student;
- Submission status of the weekly assignments;
- Study plan—regular or procrastinated;
- Stress level as assessed by the student.

**Figure 2:**
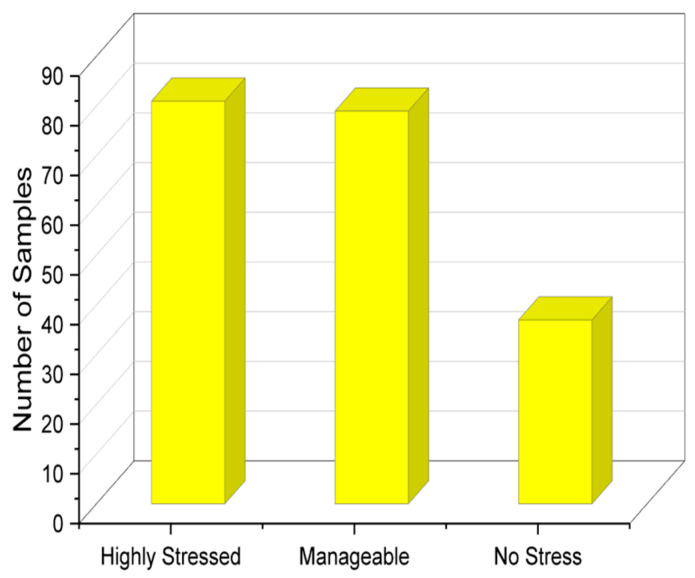
Stress level distribution of students.

The variables that are categorical are as follows: most productive time, submission status of weekly assignments, study plan, and stress level. The remaining variables are numeric; 197 responses were collected from the community, and exploratory analysis of the results was performed to analyze the responses. In the given problem statement, we have multiple columns representing different variables related to student stress levels. We performed the Tukey test [30], which allows for multiple comparisons between the levels of each variable, providing valuable insights into the differences and relationships among these variables. This capability is particularly useful when trying to identify which variables have a significant impact on the stress levels of students. On top of this, the test is known for its ability to handle small sample sizes effectively. It does not rely on strict assumptions about the underlying data distribution, making it suitable for datasets with relatively limited observations. The number of samples with respect to each stress class is analyzed in Figure 2. It shows that most students were highly stressed or were able to manage stress; however, a fraction of the students answered by saying that they experienced no stress as well.

The average sleeping hours of a student fall more or less between the range of 6 to 8 h, which is a healthy sign; however, there is still a fraction of people sleeping fewer hours than ideal, which is not good for our bodies. It can be seen that the people who are observed to be highly stressed have a sleep time of less than the ideal amount of 6 h a day.

From Table 3, it is inferred that out of the 197 responses, 144 students sleep for at least a minimum of six hours, which is the recommended minimum sleeping time for a person. It also shows that the people who sleep for less than 6 h are more likely to be highly stressed than not. The data for highly stressed students can be seen toward the top left of the table, which implies that there is a correlation between sleep hours and stress levels. The absolute value for the highly stressed might be higher in the 6 h range, but this is because there are more entries, i.e., more people who achieve around 6 h of sleep compared to the other data points.

**Figure 3:**
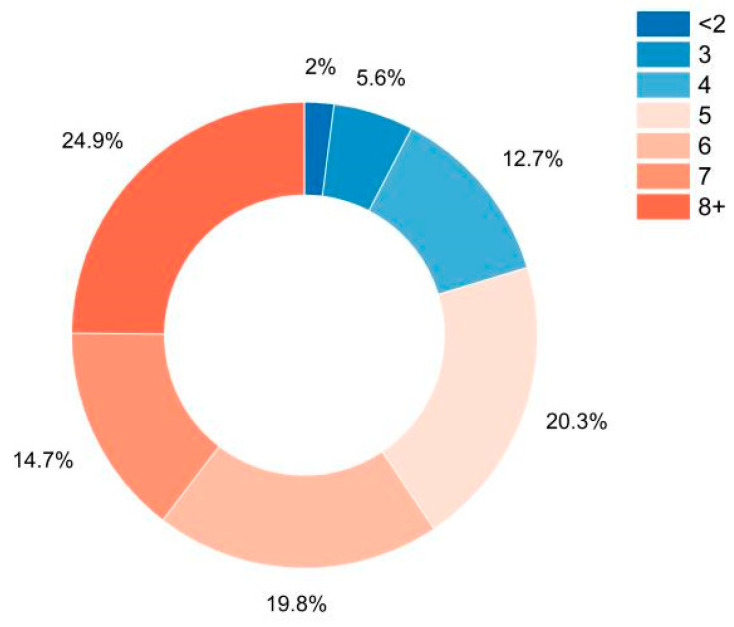
Hours of exposure to screen time.

The following figures and tables focus on how screen time plays an integral part in determining the stress levels of students. Figure 3 shows a donut graph that displays the percentage of students exposed to screen time on their phones or laptops.

Table 4 compares the screen time of the students and the number of weekly assignments they receive, and from it, we can see that many people who obtain more than five weekly assignments have an alarming screen time of more than 8 h a day.

It is evident that the amount of screen time has shot up considerably, considering that the classes for the students have shifted online, and nearly 160 students have screen time greater than or equal to 5 h. Table 5 shows how the stress levels are impacted with respect to the number of weekly assignments given to the students with respect to each stress level classification.

It can be inferred that most of the students manage stress as long as the assignments per week are fewer than or equal to 3, and it as goes beyond 4, the stress levels become higher, indicating that the students find it difficult to manage the workload of the academics. Table 6 shows the representation of how sleep hours are affected with respect to the productive time period of each student.

**Figure 4:**
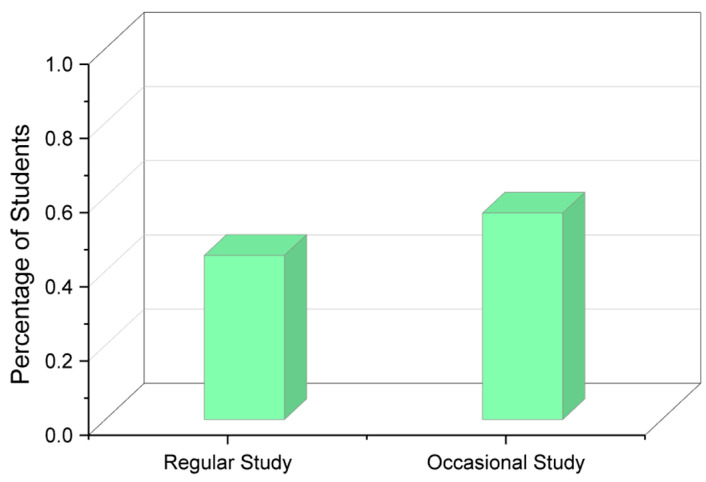
Studying methodology of students.

It is clearly visible that the students who sit up late at night tend to sleep for fewer hours than the ones who are productive early in the morning. The late-night workers tend to sleep as little as 4 h, while the ones who wake up early in the morning obtain at least a minimum of 6 h of sleep. Figure 4 shows which methods students adopt when it comes to studying, and more than half the responses state that the students study occasionally rather than regularly.

### 5.2. Data Preprocessing

After the exploratory analysis is completed, data preprocessing is performed to prepare the data for use in training machine learning models. Symbols such as +, <, and > are removed by using the inbuilt libraries and packages in R, as explained in the algorithm section. An important step in the process is to remove the imbalance in the dataset. Table 7 shows the number of responses under each class of stress level.

**Figure 5:**
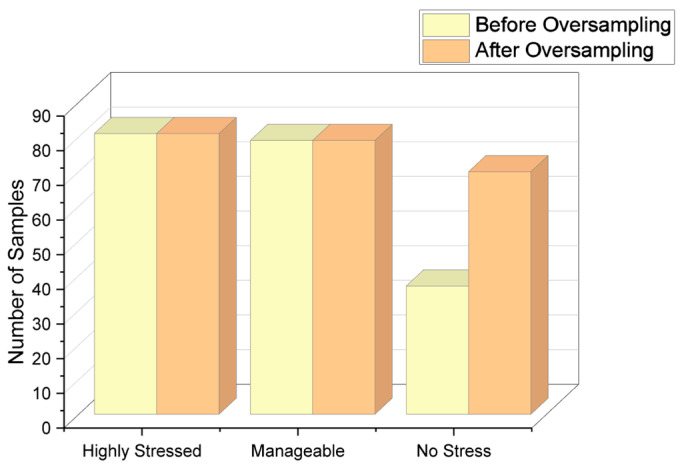
Responses after applying oversampling technique.

Since there is a large number of responses for “Highly stressed” and “Manageable” stress levels, the oversampling method has been used to bring the count of the “No stress” class closer to the count of the other two majority classes. The count of each class after applying the oversampling method is shown in Table 8. Figure 5 shows the visual representation of responses before and after applying the oversampling technique.

### 5.3. Evaluation Metrics

To evaluate the performance of each algorithm we have used by the same methods, we have chosen confusion matrices as the best way to move forward as they directly provide a numerical representation of what was predicted compared to its actual value. In a classification consisting of more than two predictable values, the confusion matrix is a *k* × *k* matrix, where *k* is the number of possible predictable values. The main diagonal of the confusion matrix shows the count of the right predictions, and the other elements show the wrong predictions. Consider the *k* × *k* confusion matrix to be *X* and each element of the matrix to be *x_ij_*. The accuracy of the algorithm can be calculated as in Equation (12).

(12) $$\;Accuracy=\frac{\sum_{i=0}^{k}\;\;x_{ii}}{N}$$

where *N* represents the total number of predicted values, and *x_ii_* are the true positives, i.e., the values in the main diagonal. Accuracy can also be represented as the following Equation (13):

(13) $$Accuracy=\frac{\mathrm{True}\;\mathrm{Positives}+\mathrm{True}\;\mathrm{Negatives}}{\mathrm{True}\;\mathrm{Positives}+\mathrm{False}\;\mathrm{Negatives}+\mathrm{True}\;\mathrm{Negatives}+\mathrm{False}\;\mathrm{Positives}}$$

The other metric that is useful to analyze the performance of an algorithm is the F1 score, which is defined as the harmonic mean of precision and recall. Here, precision quantifies the number of positive class predictions that actually belong to the positive class, and recall quantifies the number of positive class predictions formulated out of all positive examples in the dataset, and these are described in Equations (14)–(16).

(14) $$Recall=\frac{\mathrm{True}\;\mathrm{Positives}}{\mathrm{True}\;\mathrm{Positives}+\mathrm{False}\;\mathrm{Negatives}}$$

(15) $$Precision=\frac{\mathrm{True}\;\mathrm{Positives}}{\mathrm{True}\;\mathrm{Positives}+\mathrm{False}\;\mathrm{Positives}}$$

(16) $$F1\;score=2\;*\;\frac{Precision\;*\;Recall}{Precision+Recall}$$

## 6. Results

### 6.1. Statistical Test Results

Two statistical tests, the analysis of variance (ANOVA) and the Tukey test, have been implemented and analyzed on the data. The purpose of the tests is to check if the statistical analysis proceeds to show that there exists a difference between the means of different populations, i.e., the stress-level classes in this scenario. The numerical variables, namely the sleep hours, the number of assignments, and the average screentime of students, have been taken into consideration for the tests to check the impact of the factors. A one-way ANOVA test was initially conducted using the three parameters, keeping the stress-level classes as the factors. Table 9 shows the F score for each parameter with respect to the factor variable.

It was inferred that there was a significant difference between the population at the 0.05 significance level when tested with all three numeric parameters. Following the one-way ANOVA test, a Tukey test was conducted on the dataset to analyze the difference in means for the parameters with respect to the factor variable.

When tested with the sleep hour parameter, it was inferred that there was a significant mean difference in those who were highly stressed but no difference between those who managed the stress well or were under no stress. Table 10 and Table 11 show the Tukey test mean comparisons by taking the sleep hours parameter into consideration.

When the test was conducted using the assignment parameter, it was inferred that there existed a significant difference between all three groups. Table 12 and Table 13 show the Tukey test mean comparison by taking the assignment parameter into consideration.

When tested with the screen-time parameter, it was inferred that there was a significant mean difference in those who were highly stressed but no difference between those who managed the stress well or were under no stress. Table 14 and Table 15 show the Tukey test mean comparison by taking the sleep hours parameter into consideration.

### 6.2. Experimental Results

**Figure 6:**
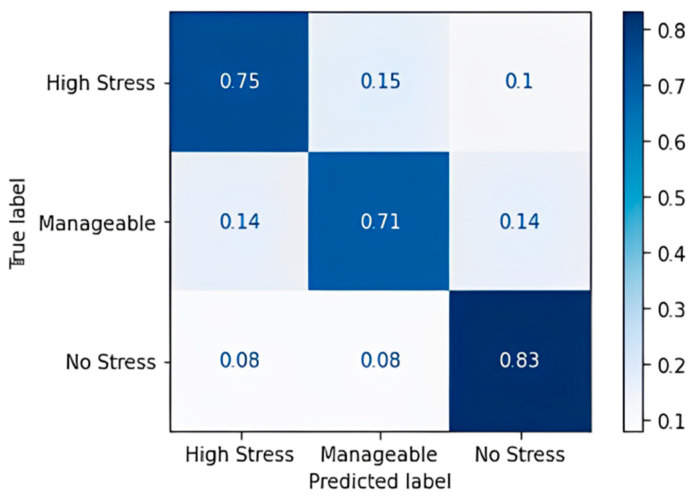
Confusion matrix accuracy for decision tree classifier.

The data are split into 80% training data and 20% testing data and provided to various ML algorithms to classify the stress levels. From the decision tree obtained, it was observed that those who study regularly are under no stress—even more so when the assignments given to them per week are fewer than or equal to 4. As the assignments keep increasing, the screen time increases and goes up to 7 or 8 h as well, and that, in turn, has led to the prediction of the class to be “Highly stressed”, while other cases are mostly predicting manageable stress levels. Table 16 shows the confusion matrix obtained for the decision tree classifier, and Figure 6 shows the accuracy in terms of the classification percentage.

**Figure 7:**
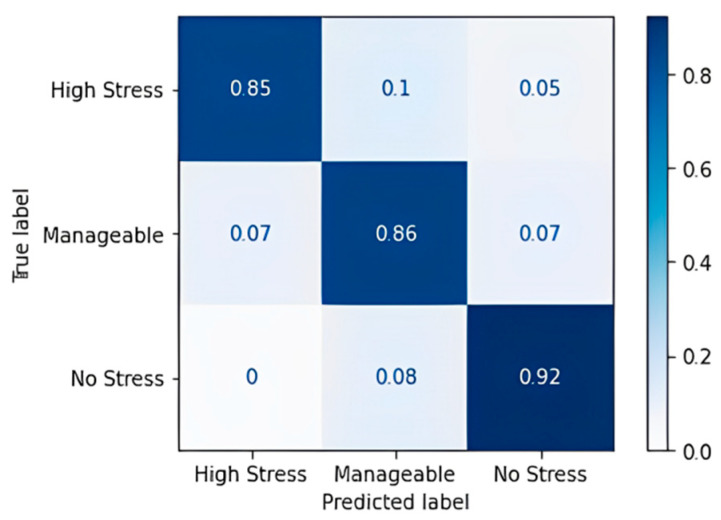
Confusion matrix accuracy for random forest classifier.

For the most part, the algorithm has predicted correctly, except for a few cases of the high-stress class for which it predicted “Manageable” and “No Stress”. A classification accuracy of 76.09% and an F1 score of 75.67% were obtained upon applying the decision tree classification algorithm. Table 17 shows the confusion matrix obtained for the random forest algorithm, and Figure 7 shows the accuracy in terms of the classification percentage.

**Figure 8:**
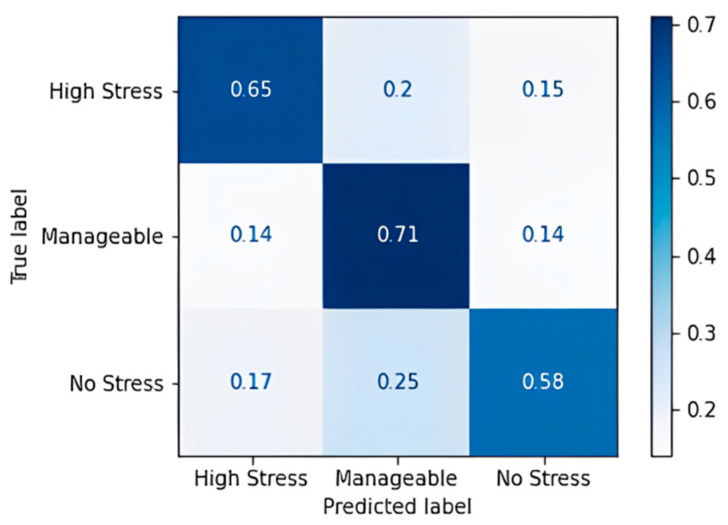
Confusion matrix accuracy for gradient boost algorithm.

The random forest algorithm performs better in comparison to the decision tree. Noticeable wrong predictions are in the high and manageable stress classes. An accuracy of 86.96% and an F1 Score of 86.67% were obtained, which implies that the random forest model has more true positives and true negatives when it classifies and a smaller number of false positives and false negatives than the decision tree model that we obtained. Table 18 shows the confusion matrix obtained for the gradient boost algorithm, and Figure 8 shows the accuracy in terms of the classification percentage.

**Figure 9:**
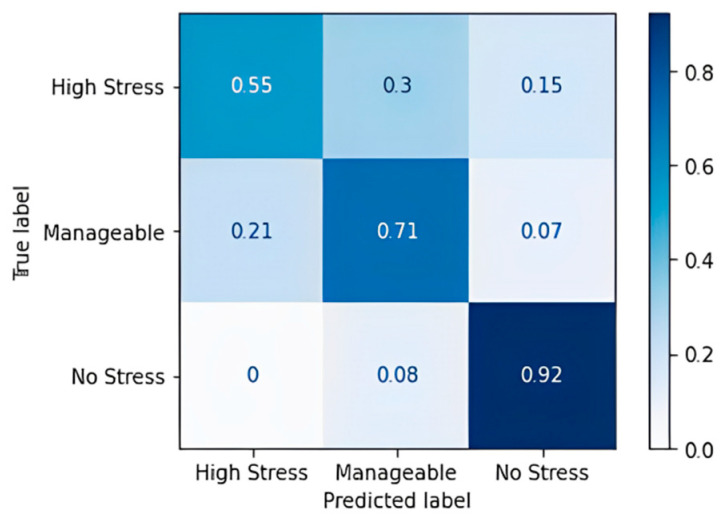
Confusion matrix accuracy for AdaBoost algorithm.

The gradient boost algorithm has some imperfections in predicting all three classes. The gradient boost model was trained with the multinomial loss function, and an accuracy of 65.22% and an F1 score of 64.3% were obtained. Table 19 shows the confusion matrix obtained for the AdaBoost algorithm, and Figure 9 shows the accuracy in terms of the classification percentage.

The AdaBoost algorithm performs a little better than the gradient boost algorithm. The algorithm yielded results with an accuracy of 69.57% and an F1 score of 70.3%.

### 6.3. Comparison of Algorithms

**Figure 10:**
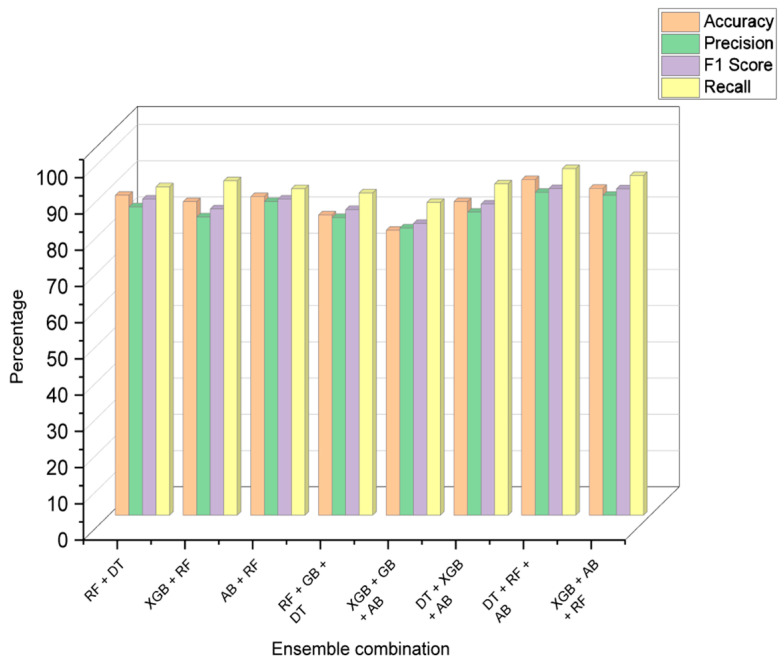
Comparison of ensemble learning performance metrics.

After analyzing the performance of each model individually, a combination of models was used to predict the stress level. The ensemble learning methods were judged by the combined performance of accuracy, precision, F1 score, and recall. Table 20 and Table 21 consist of the results of the performance metrics of the decision models and ensemble learning models, respectively, and Figure 10 shows the visual comparison of the evaluation metrics of each combination of ensemble models.

**Figure 11:**
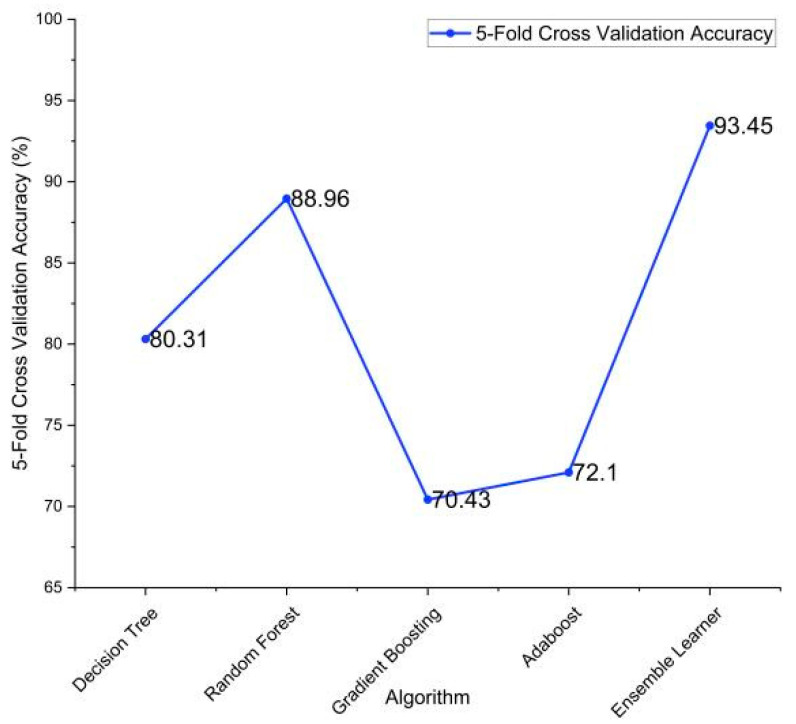
Fivefold cross-validation accuracies of the implemented algorithms.

In the table, DT—decision tree, RF—random forest, XGB—XG boost classifier, and AB—AdaBoost classifier. The combination of DT + RF + AB provided the best results with an accuracy of 93.48%. The confusion matrix of this is listed below in Table 22. This essentially highlights the importance of using ensemble methodology to improve the overall performance. Fivefold cross-validation was performed on the individual models and the ensemble model, and the results are described in Table 23 and visualized in Figure 11.

**Figure 12:**
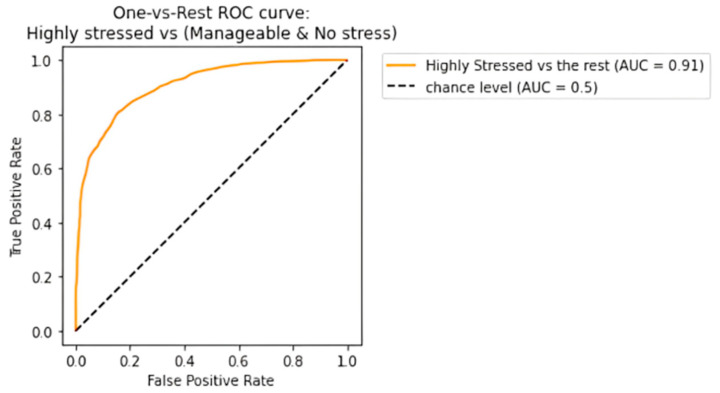
ROC-AUC curve for high-stress class.

**Figure 13:**
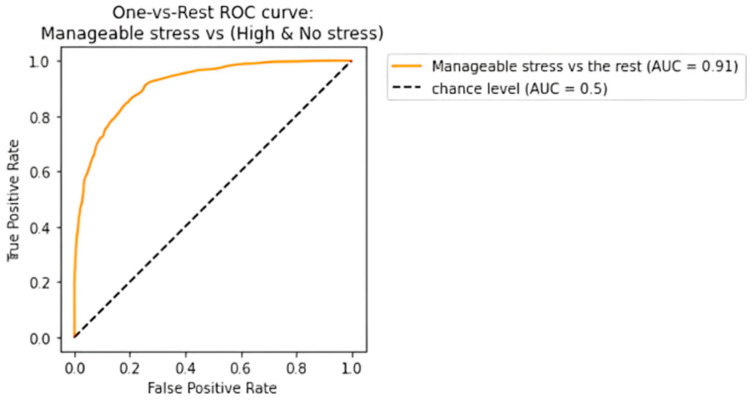
ROC-AUC curve for manageable-stress class.

**Figure 14:**
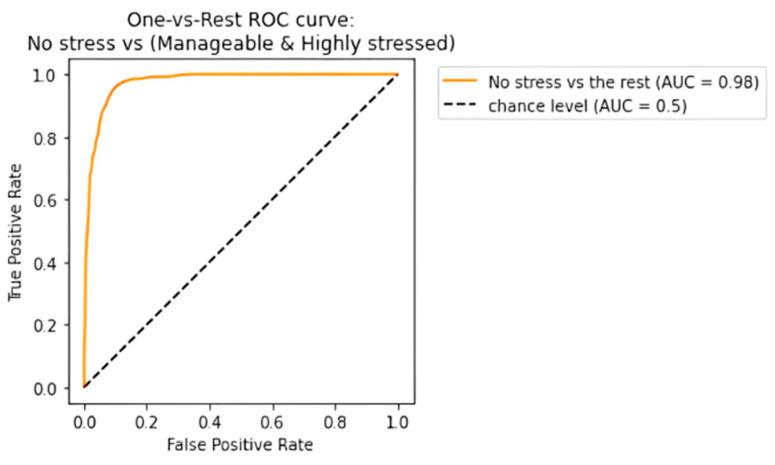
ROC-AUC curve for no-stress class.

The receiving operating characteristic (ROC) curve has been plotted for the efficient ensemble learning algorithm (DT + RF + AB), which provided the best results. Figure 12, Figure 13 and Figure 14 show an area under the curve (AUC) for each predicted class. The high-stress and manageable-stress categories had true-positive rates of 91%, while the no-stress category had a rate of 98%.

**Figure 15:**
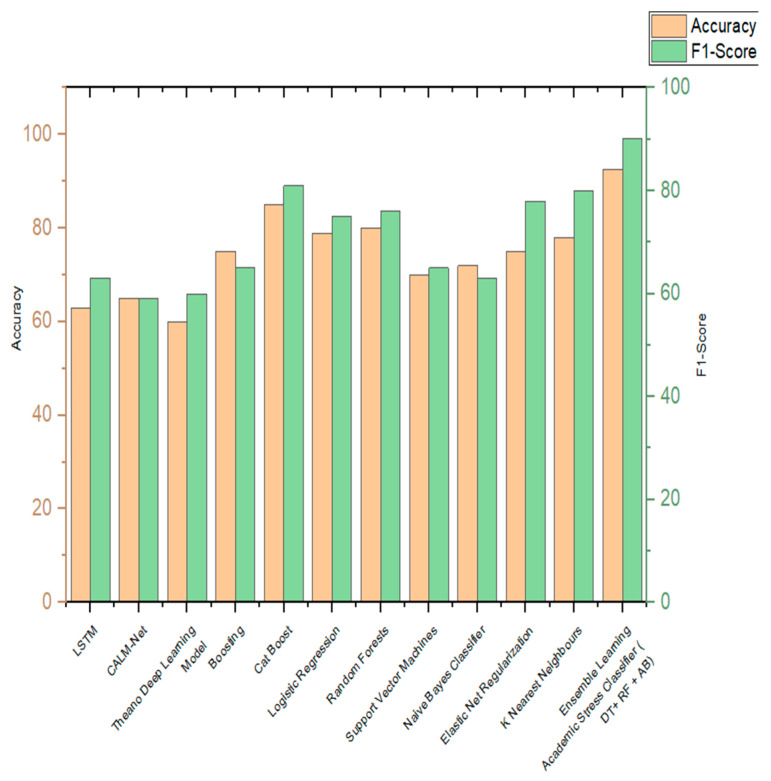
Comparison graph of proposed work with other existing works.

The efficient ensemble learning stress classifier developed in this paper has been compared with the various state-of-the-art algorithms. Table 24 shows the comparisons of algorithms incorporated by various authors in the classification of stress and is being compared to the ensemble learning academic stress classifier implemented in this paper. Figure 15 shows the visual representation of the same. The parameters of comparison are accuracy and F1 score.

## 7. Discussion and Future Work

This paper analyzes surveyed data in order to classify the stress levels of a student based on different machine learning models. To remove the imbalance in the minority class, an oversampling method was used to sample responses with “no stress” classification. To measure the performance of the models, confusion matrix, accuracy, precision, recall, and the F1 score was used. Based on the results, the ensemble learning academic stress classifier provided the best results with an accuracy of 93.48% and an F1 score of 93.14%. Fivefold cross-validation was also performed, and an accuracy of 93.45% was achieved. The individual machine learning models also provided good results with random forest alone achieving an accuracy of 88.96%. This highlights the importance of enhanced prediction using the ensemble learning techniques. As a result of this analysis, students are able to gain insights into where their daily habits can be improved in order to maintain a stress-free academic lifestyle.

Importantly, this research extends beyond the immediate results and paves the way for further exploration in the field of stress prediction and mitigation. Previous studies have delved into stress prediction across various domains, offering valuable insights into the broader landscape of human well-being. However, our work distinguishes itself by focusing exclusively on students and their academic stressors, shedding light on a crucial facet of stress prediction. By narrowing the scope to academic factors, we bridge the gap in existing research, ensuring that the unique stressors faced by students are comprehensively addressed. The future work involves analyzing patterns of the responses across different classes in terms of the stress level and developing a personalized assistant where students can enter the value for each attribute and obtain strategies that may help them improve their academic performances based on the predictions that the model provides, and the model will be able to learn from the responses of the students with lower stress levels. It is also possible to perform conventional statistical analysis with the dependent variable as stress and keeping the predictors as other variables. Analyses such as binary logistic regression, method enter, and method backward conditional can be performed. With the advancement of technology, electronic devices have become an integral part of our daily lives, providing an opportunity to gather valuable information without direct user involvement. By incorporating sensors or logging mechanisms into electronic devices such as smartphones or smartwatches, it is possible to passively collect data on various factors related to student stress levels, such as physical activity, screen time, location, and communication patterns. This passive data-collection approach eliminates the need for manual data entry through forms, reducing the potential for self-reporting biases and enhancing the ecological validity of the dataset. In this way, students can receive better insights into how to advance academically while keeping stress levels in check. The broader implications of this work resonate with the concept of predictive analytics for stress management, not only within the academic realm but also across diverse domains. Our findings underscore the potential for a personalized assistant that allows students to input their attributes and receive tailored strategies to enhance their academic performances, leveraging the predictive capabilities of our model. This approach presents an innovative paradigm in stress mitigation, one that learns from the experiences of students with lower stress levels, thereby contributing to the development of more effective stress management techniques.

## Figures and Tables

**Table 1:** Key contributions and algorithms used in different fields of scope.

S. No.	Scope	Works	Key Contributions	Algorithms Used
1.	Deep Learning	Acikmese, Y., et al. (2019) [6]	Worked on the StudentLife project, which collects passive and automatic data from students’ phones in Dartmouth.	Recurrent Neural Networks using LSTMs
		Shaw, A. (2019) [7]	Worked on personalized stress prediction of students in SmartLife study in Dartmouth.	Deep Multitask Network using LSTMs
		Wang, R. (2014) [8]	Worked on StudentLife dataset and analyzed stress factors	LSTMs and Correlation of factors
		Raichur, N., et al. (2017) [9]	Detected stress from facial expressions presented in an image of subjects.	Deep learning models using theano package in python and linear regression
2.	Supervised and Ensemble Learning	Reddy, U. S., et al. (2018) [10]	Predicted stress levels in working employees from the OSMI Mental Health in Tech 2017 dataset.	Logistic Regression, KNN, Decision Tree, Random Forest, Bagging, Boosting
		Gamage, S. N., et al. (2022) [11]	Predicting mental distress in the IT workforce during the height of the pandemic in a remote environment.	Random Forests, SVM, XGBoost, CatBoost, Decision Tree, Naïve Bayes
		Rahman, A. A., et al. (2022) [12]	Analysis of stress in undergraduate students from Jordan at the height of the pandemic.	Linear Regression, Logistic Regression
		Priya, A., et al. (2020) [14]	Detected anxiety, depression, and stress using DASS 21.	Decision Trees, Random Forests, Naïve Bayes. SVMs, KNNs
		Rois, R., et al. (2021) [15]	Researched causes of stress in Bangladesh university students.	Decision Tree, Random Forest, SVM, Logistic Regression
		Jaques, N., et al. (2015) [16]	Modeled students’ happiness by monitoring their phones.	SVMs, Random Forests, Logistic Regression, KNN, Naïve Bayes, Adaboost.
		Flesia, L., et al. (2020) [17]	Predicted perceived stress on adults caused by the pandemic.	SVMs, Logistic Regression, Random Forests, Naïve Bayes
		Li, H., et al. (2022) [18]	Predicted perceived stress based on micro-EMA history data of adults.	Elastic Net Regression, Super Vector Regression, Gradient Boosted Regression Trees
		Pabreja, K., et al. (2021) [19]	Predicted stress in students transitioning from adolescent teens to adults.	Random Forests
		Bisht, A., et al. (2022) [20]	Analyzed stress in over 190 school kids aged 14–18.	Decision Trees, Logistic Regression, KNN, Random Forest
		Martino, F. D., et al. (2020) [21]	Developed a tool to predict high-resolution stress for health systems to aid in clinical treatments.	Random Forests, Least-Squares Gradient Boosting, Nonlinear AutoRegressive Network
3.	Stress Factors Analysis	Pariat, M. L., et al. (2014) [22]	Tried to find relationship between stressors.	Logistic Regression, Correlation
		Kim, J., et al. (2014) [23]	Analyzed the impact of exercise on stress and mental well-being of university students.	Correlation
		Pryjmachuk, S., et al. (2007) [24]	Predicted stress in nursing students from a top English university.	Logistic Regression, Correlation.

**Table 2:** Depiction of stress parameters considered in various works.

S. No.	Scope	Works	Stress Parameters			
			S1	S2	S3	S4
1.	Deep Learning	Acikmese, Y., et al. (2019) [6]	Yes	No	No	No
		Shaw, A. (2019) [7]	Yes	No	No	No
		Wang, R. (2014) [8]	Yes	No	No	No
		Raichur, N., et al. (2017) [9]	Yes	No	No	No
	Supervised and Ensemble Learning	Reddy, U. S., et al. (2018) [10]	No	Yes	Yes	No
		Gamage, S. N., et al. (2022) [11]	No	Yes	Yes	No
		Rahman, A. A., et al. (2022) [12]	Yes	No	No	Yes
		Priya, A., et al. (2020) [14]	No	No	Yes	No
		Rois, R., et al. (2021) [15]	Yes	No	Yes	No
		Jaques, N., et al. (2015) [16]	Yes	No	Yes	No
		Flesia, L., et al. (2020) [17]	No	No	Yes	Yes
		Li, H., et al. (2022) [18]	No	No	Yes	Yes
		Pabreja K., et al. (2021) [19]	Yes	No	Yes	No
		Bisht, A., et al. (2022) [20]	Yes	No	Yes	Yes
		Martino, F. D., et al. (2020) [21]	No	No	Yes	No
3.	Analysis of Stress Factors	Pariat, M. L., et al. (2014) [22]	Yes	No	Yes	No
		Kim, J., et al. (2014) [23]	Yes	No	Yes	No
		Pryjmachuk, S., et al. (2007) [24]	Yes	No	Yes	No

**Table 3:** Analysis of stress with sleeping hours of students.

Sleep (Hours)	Highly Stressed	Manageable	No Stress
<=4	23	3	0
5	17	6	4
6	23	35	5
7	12	24	11
>=8	6	11	17

**Table 4:** Screen time on a daily basis vs. weekly assignments.

Weekly Assignments (Count)/Screen Time (Hours)	1 h	2 h	3 h	4 h	5+ h
2	1	0	2	0	1
3	0	7	1	1	2
4	4	8	9	3	1
5	5	8	21	5	1
6	3	6	9	11	10
7	2	2	5	9	11
8+	3	7	7	10	22

**Table 5:** Impact of assignments on stress levels of the students.

Assignments	Highly Stressed	Manageable	No Stress
1	8	4	6
2	14	10	14
3	5	36	12
4	19	18	2
5+	34	11	3

**Table 6:** Analyzing impact of sleep hours on the productivity of a student.

Sleep (Hours)	Early Morning Risers	Late-Night Workers
4	4	21
5	5	22
6	27	36
7	21	26
8+	23	11

**Table 7:** Classification of stress levels in initial data response.

Highly Stressed	Manageable	No Stress
81	79	37

**Table 8:** Classification of stress-level responses after oversampling.

Highly Stressed	Manageable	No Stress
81	79	70

**Table 9:** F-score value for each parameter in a one-way ANOVA test.

Parameter	F Score
Sleep	16.25
Assignments	17.09
Screentime	8.48

**Table 10:** Tukey test mean comparison grouping letters table with respect to sleep hours.

Stress Level	Mean	Groups
Highly stressed	3.19753	A
No stress	2.37838	B
Manageable	2.10127	B

**Table 11:** Tukey test mean comparison with respect to sleep hours.

Category	MeanDiff	SEM	q Value	Prob	Alpha	Sig	LCL	UCL
Manageable/Highly Stressed	0.91186	0.17409	7.40733	<0.0001	0.05	1	0.50068	1.32304
No Stress/Highly Stressed	1.58959	0.21846	10.29021	<0.0001	0.05	1	1.07362	2.10556
No Stress/Manageable	0.67773	0.21933	4.36997	0.00647	0.05	0	0.15971	1.19574

**Table 12:** Tukey test mean comparisons grouping letters table with respect to assignments.

Stress Level	Mean	Groups
No stress	3.86486	A
Manageable	3.20253	B
Highly stressed	2.34568	C

**Table 13:** Tukey test mean comparison with respect to assignments.

Category	MeanDiff	SEM	q Value	Prob	Alpha	Sig	LCL	UCL
Manageable/Highly Stressed	−0.42522	0.192	3.13214	0.07121	0.05	1	−0.87869	0.02824
No Stress/Highly Stressed	−1.19019	0.24093	6.9863	<0.0001	0.05	1	−1.75922	−0.62116
No Stress/Manageable	−0.76497	0.24188	4.47257	0.00514	0.05	1	−1.33625	−0.19368

**Table 14:** Tukey test mean comparison grouping letters table with respect to screen time.

Stress Level	Mean	Groups
No stress	3.75676	A
Manageable	3.34177	A
Highly stressed	2.53086	B

**Table 15:** Tukey test mean comparison with respect to screen time.

Category	MeanDiff	SEM	q Value	Prob	Alpha	Sig	LCL	UCL
Manageable/Highly Stressed	−0.90999	0.24621	5.22682	8.29298 × 10^−4^	0.05	1	−1.4915	−0.32847
No Stress/Highly Stressed	−1.33934	0.30896	6.13056	<0.0001	0.05	1	−2.06906	−0.60962
No Stress/Manageable	−0.42935	0.31019	1.95753	0.35117	0.05	0	−1.16196	0.30326

**Table 16:** Confusion matrix for decision tree algorithm.

Label	Highly Stressed	Manageable	No Stress
Highly Stressed	15	3	2
Manageable	2	10	2
No Stress	1	1	10

**Table 17:** Confusion matrix for random forest algorithm.

Label	Highly Stressed	Manageable	No Stress
Highly Stressed	17	2	1
Manageable	1	12	1
No Stress	0	1	11

**Table 18:** Confusion matrix for gradient boost algorithm.

Label	Highly Stressed	Manageable	No Stress
Highly Stressed	13	4	3
Manageable	2	10	2
No Stress	2	3	7

**Table 19:** Confusion matrix for AdaBoost algorithm.

Label	Highly Stressed	Manageable	No Stress
Highly Stressed	11	6	3
Manageable	3	10	1
No Stress	0	1	11

**Table 20:** Performance metrics of decision models.

S. No	Algorithm	Accuracy	F1 Score
1.	Decision Tree	76.09%	75.67%
2.	Random Forest	86.96%	86.67%
3.	Gradient Boost	65.22%	64.3%
4.	AdaBoost	69.57%	70.3%

**Table 21:** Performance metrics of ensemble models.

S. No	Ensemble Combination	Accuracy	Precision	F1 Score	Recall
1.	RF + DT	88.34%	85.12%	87.26%	90.67%
2.	XGB + RF	86.57%	82.35%	84.55%	92.34%
3.	AB + RF	87.96%	86.61%	87.26%	90.14%
4.	RF + GB + DT	82.90%	82.17%	84.39%	88.96%
5.	XGB + GB + AB	78.67%	79.29%	80.54%	86.34%
6.	DT + XGB + AB	86.57%	83.65%	85.89%	91.47%
7.	DT + RF + AB	93.48%	92.99%	93.14%	93.30%
8.	XGB + AB + RF	90.23%	88.32%	90.11%	93.76%

**Table 22:** Confusion matrix of ensemble algorithm DT + RF + AB.

Label	Highly Stressed	Manageable	No Stress
**Highly Stressed**	18	1	0
**Manageable**	0	13	1
**No Stress**	0	1	12

**Table 23:** Fivefold cross-validation accuracies.

Algorithm	Fivefold Validation Accuracy
Decision Tree	80.31%
Random Forest Classifier	88.96%
Gradient Boosting	70.43%
AdaBoost Algorithm	72.1%
Ensemble Learning (DT + RF + AB)	93.45%

**Table 24:** Comparison results of proposed work and other works.

S. No.	Work	Algorithm	Accuracy	F1 Score
1.	Acikmese, Y., et al. (2019) [6]	LSTM	63%	63%
2.	Shaw, A. (2019) [7]	CALM-Net	65%	59%
3.	Raichur, N., et al. (2017) [9]	Theano Deep Learning Model	60%	60%
4.	Reddy, U. S., et al. (2018) [10]	Boosting	75%	65%
5.	Gamage, S. N., et al. (2022) [11]	Cat Boost	85%	81%
6.	Rahman, A. A., et al. (2022) [12]	Logistic Regression	79%	75%
7.	Rois, R., et al. (2021) [15]	Random Forests	80%	76%
8.	Jaques, N., et al. (2015) [16]	Support Vector Machines	70%	65%
9.	Flesia, L., et al. (2020) [17]	Naïve Bayes Classifier	72%	63%
10.	Li, H., et al. (2022) [18]	Elastic Net Regularization	75%	78%
11.	Bisht, A., et al. (2022) [20]	K Nearest Neighbours	78%	80%
12.	Ensemble Learning Academic Stress Classifier	DT + RF + AB	93.48%	93.14%

## Data Availability

Data are contained within the article.
